# Factors associated with general practitioners’ awareness of depression in primary care patients with heart failure: baseline-results from the observational RECODE-HF study

**DOI:** 10.1186/s12875-017-0641-1

**Published:** 2017-06-09

**Authors:** Marion Eisele, Anja Rakebrandt, Sigrid Boczor, Agata Kazek, Nadine Pohontsch, Magdalena Okolo-Kulak, Eva Blozik, Jens-Martin Träder, Stefan Störk, Christoph Herrmann-Lingen, Martin Scherer, for the RECODE Study Group, Winfried Adam, Cassandra Behrens, Eva Blozik, Sigrid Boczor, Marion Eisele, Malte Harder, Christoph Herrmann-Lingen, Agata Kazek, Dagmar Lühmann, Anja Rakebrandt, Koosje Roeper, Martin Scherer, Stefan Störk, Jens-Martin Träder

**Affiliations:** 1 Department of Primary Medical Care, Center for Psychosocial Medicine, University Medical Center Hamburg-Eppendorf, Martinistraße 52, 20246 Hamburg, Germany; 2 Department of Primary Medical Care, University of Luebeck, Ratzeburger Allee 160, 23538 Luebeck, Germany; 3 Comprehensive Heart Failure Center Würzburg, University and University Hospital Würzburg, Straubmühlweg 2a, 97078 Würzburg, Germany; 4 University of Göttingen Medical Center and German Center for Cardiovascular Research, partner site Göttingen, von-Siebold-Str. 5, D-37099 Göttingen, Germany

**Keywords:** Depression, Heart failure, Recognition of depression, Observational study, Primary care, Health care research

## Abstract

**Background:**

Depression is more prevalent in patients with heart failure (HF) than in those without, but its detection is complicated by the symptom overlap between the two diseases. General practitioners (GPs) are the first point of contact for patients with HF. Therefore, this study aims to investigate GPs’ awareness of depression in their HF patients and factors associated with this awareness.

**Methods:**

In this cross-sectional, observational study 3224 primary care patients with HF were screened for depressive symptomatology using an algorithm based on the Hospital Anxiety and Depression Scale, the 9-item subscale on Depression of the Patient Health Questionnaire, and selected items from the PROMIS Depression and Anxiety scales. The 272 GPs of all patients involved in the study were interviewed by telephone regarding their patients’ somatic and psychological comorbidities. The awareness rates of depressive symptomatology by the patients’ GPs are analyzed using descriptive statistics. Logistic regression analyses are applied to investigate the patient- and GP-based factors associated with the GPs’ awareness of depressive symptomatology.

**Results:**

GPs were aware of their patients’ depressive symptomatology in 35% of all cases. Factors associated with the awareness of depressive symptomatology were: higher patient education levels, a history of depression known to the GP, GP-consultations due to emotional distress within the last 6 months, a higher frequency of GP-contacts within the last 6 months, a higher New York Heart Association (NYHA) classification and more severe depressive symptomatology. The GPs’ characteristics, including further education in psychology/psychiatry, were not associated with GP awareness.

**Conclusions:**

Many aspects, including the definition of awareness and the practical issues in primary care, may contribute to the unexpectedly low awareness rates of depressive symptomatology in HF patients in primary care. Awareness rates might increase, if GPs encouraged their patients to talk about emotional distress, held detailed medical interviews including a patient’s history of depression and payed special attention to HF patients with low education levels. However, it remains to be investigated whether GPs’ judgement of depressive symptomatology is a better or worse indicator for the future prognosis and quality of life of HF patients than psychiatry based diagnostic criteria.

## Background

Chronic heart failure (HF) is a disease with high rates of mortality and hospital admissions [1, 2]. In 2013 61.7 million people suffered from HF worldwide, 55% of those at a severe stage [3]. Depression is more prevalent in HF patients than in those without HF, probably because severe HF symptoms can affect a person’s functional status, trigger anxiety and depression [4, 5], and, thus, impair a patient’s health-related quality of life [6–8]. At the same time depression reduces a patient’s ability to cope with physical symptoms and adhere to medical treatment [9] which in turn leads to poorer outcomes in HF treatment, including higher rates of mortality and hospital admissions [10]. The detection of depression is especially complicated in patients with HF due to the symptom-overlap between HF and depression (e.g. fatigue, listlessness, and sleep disturbance). Because the symptom overlap exacerbates detecting depression, national and international HF guidelines recommend the regular and systematic assessment of depression during the diagnostic process and treatment of HF [11–15]. However, there is no current evidence demonstrating the benefits of depression screening in chronic diseases [16].

If patients with depression seek medical treatment, most consult a primary care physician (GP) [17]. A positive screening for depression in primary care has shown that HF patients have a considerably higher likelihood of receiving mental health treatment [9], hence GPs play a key role in detecting potential depressions. In an international review, 47.3% of diagnosed depressions in all primary care patients were correctly identified, while the diagnostic sensitivity varied greatly between 6.6 and 78.8% [18]. Only two studies investigated the recognition of depression and the factors associated with (non-) recognition of depression in HF patients. Both studies recruited inpatients, therefore, the results are not applicable to primary care patients with HF [9, 19].

We aimed to investigate (1) to what extent the GPs were aware of their HF patients’ depressive symptomatology and (2) which factors were associated with this GPs’ awareness. We hypothesized, that awareness rates were comparably or slightly lower in primary care patients with HF when compared to all primary care patients. We further hypothesized that GPs’ awareness rates of depressive symptomatology would be associated with both patient-based and GP-based factors.

## Methods

In this cross-sectional, observational study we recruited primary care patients with HF in Germany between 2/2012 and 6/2014. Data was collected between 8/2012 and 11/2014. A detailed description of all study procedures is described in the study protocol [20].

### Recruitment

**Fig. 1:**
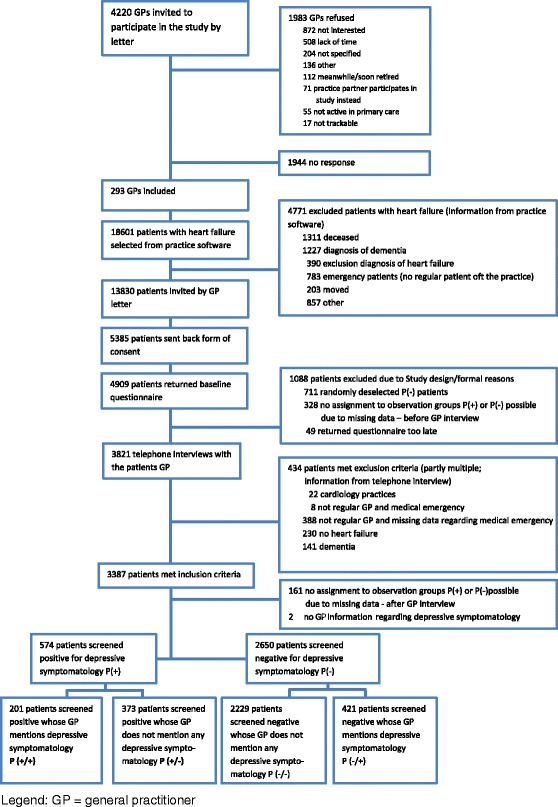
Sampling frame

The recruitment was conducted by the two study centers Hamburg (northern Germany) and Würzburg (southern Germany). All GPs in four German cities (south: Würzburg; north: Hamburg, Lübeck, Kiel) and surrounding areas received a written invitation to participate in the study and were contacted by phone, if they did not respond to the letter. Of the 4420 GPs invited, 293 were willing to participate (response rate 6.6%). The participating GPs sent written invitations to all HF patients in their practice inviting them to join in the study. Patient inclusion criteria were: an age of 18 years and over, a diagnosis of chronic HF documented within the last 5 years, and at least one GP contact within the last 6 months. Exclusion criteria were: dementia, death since the last GP-visit and HF patients who were not regular patients of the participating GP practice. Those patients who agreed to participate returned the informed, written letters of consent to the study center and in turn received a baseline questionnaire by mail. Of 13,830 patients invited to participate in the study by their GPs, 5385 (38.9%) consented to participate and 4909 (35.5%) sent back a baseline questionnaire (see Fig. 1).

### Patient questionnaire

The patient questionnaire included sociodemographic data (age, gender, education level according to the Comparative Analysis of Social Mobility in Industrial Nations (CASMIN criteria) [21], living arrangements, employment status, and type of health insurance), as well as questions regarding the frequency of GP contacts as well as if there were any GP-consultations due to emotional distress within the last 6 months. The patients were asked to disclose current medications being taken to treat depression, anxiety, agitation, sleep disturbance or “burnout”. The Enhancing Recovery in Coronary Heart Disease [ENRICHD] Social Support Instrument (ESSI) [22] was used to assess patients’ emotional support. The following instruments were included to screen for psychosocial comorbidity: the Hospital Anxiety and Depression Scale (HADS) [23, 24], the 9-item subscale for Depression in the Patient Health Questionnaire (PHQ-9) [25–27], as well as selected items from the Patient-Reported Outcomes Measurement Information System (PROMIS) Depression and Anxiety (PROMIS-D and PROMIS-A) scales [28, 29].

### Substudy to identify a case-finding algorithm

**Fig. 2:**
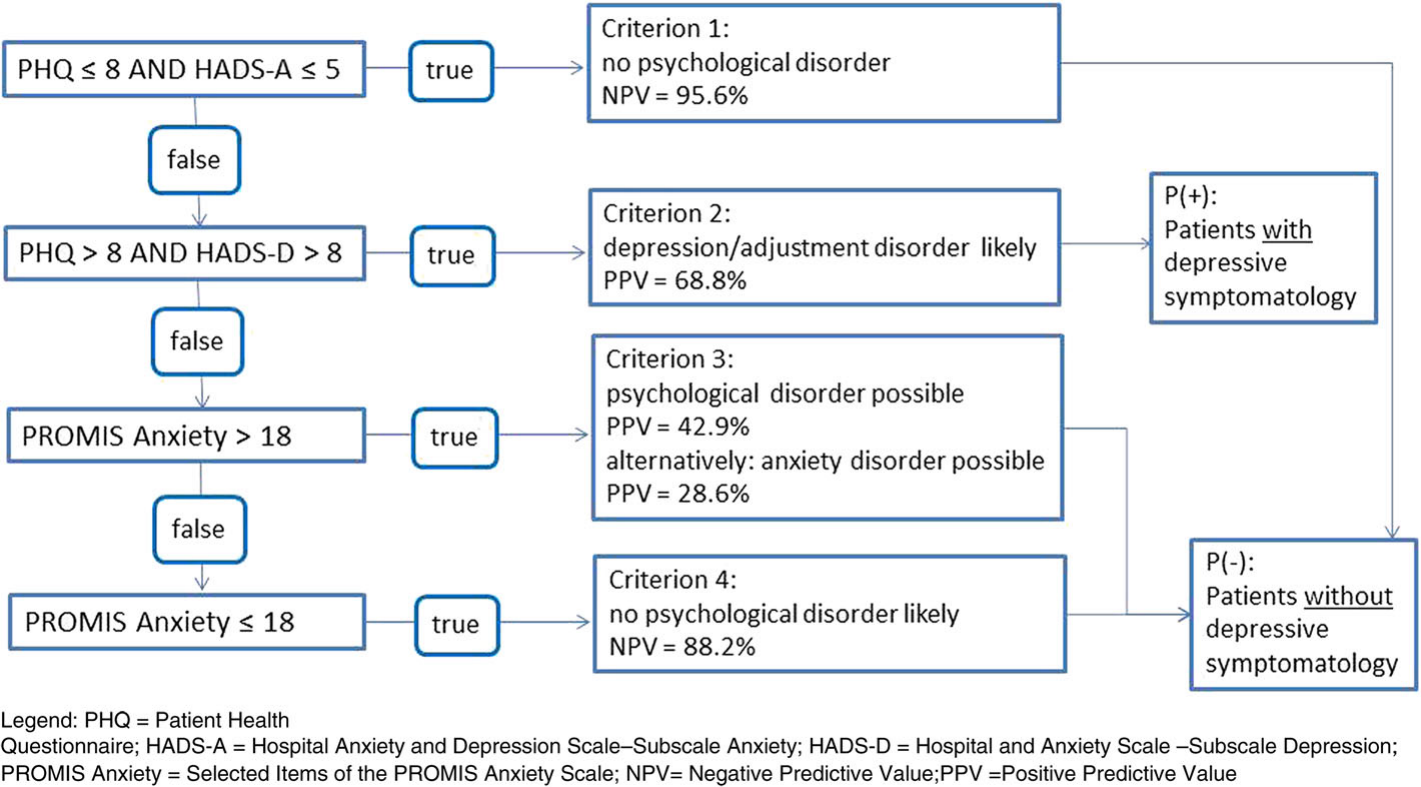
Hierarchical algorithm to identify HF patients with depression, anxiety or adjustment disorder

Due to the symptom overlap between depression and HF, a substudy was performed to establish and validate an algorithm to identify patients with depression, adjustment disorders and anxiety disorders, which is not biased by the overlapping symptoms between these disorders and HF. The aim was to identify appropriate cut-off values and combinations of established, self-rating scales to find depression and anxiety in primary care patients with HF [30]. The substudy was performed by a cooperating study center and included HF patients from an existing cohort not included in the main study. A total of 194 patients with HF completed the following questionnaires to screen for psychosocial comorbidity: HADS, PHQ-9, PROMIS-D and PROMIS-A. They additionally underwent the Structured Clinical Interview for the Diagnostic and Statistical Manual of Mental Disorders, fourth edition (SCID) [31]. The SCID is a structured, one-hour, face-to-face diagnostic interview between a trained interviewer and a patient. It was considered the gold standard for the diagnosis of disorders of the following categories: Affective disorders, anxiety disorders and adjustment disorders. Receiver operating characteristic (ROC) analyses were calculated and cut-off values were defined according to the Youden-Index, the positive predictive values (PPV) and the negative predictive values (NPV). The case finding properties have been published along with the sub-study [30]: PHQ-9 cut-off 8.5, Youden’s index 0.81; HADS-A cut-off 5.5, Youden’s index 0.53; HADS-D cut-off 8.5, Youden’s index 0.72). The Youden’s index for PROMIS 18.5 was 0.67 for any psychological disorder and 0.59 for anxiety disorder. Based on the case finding information, the following hierarchical algorithm was generated (see Fig. 2): Criterion 1 (PHQ ≤ 8 and HADS-A ≤ 5) - no psychological disorder (NPV = 95.6%); Criterion 2 (PHQ > 8 and HADS-D > 8) – depression/adjustment disorder likely (PPV = 68.8%); Criterion 3 (Criterion 1 and 2 do not apply and PROMIS Anxiety >18) – psychological disorder possible (PPV = 42.9%) [alternatively: anxiety disorder possible (PPV 28.6%)]; Criterion 4 (Criterion 1 and 2 do not apply and PROMIS Anxiety ≤18) – no psychological disorder likely (NPV = 88,2%).

### Definition of depressive symptomatology in the main study

In the main study described in this article, the algorithm was applied to all baseline questionnaires as soon as they arrived at the study center. All patients with a valid criterion 2 (depression/adjustment disorder likely) were defined as suffering from depressive symptomatology P(+). All patients with a valid criterion 3 (psychological disorder possible) remained in the study and were assigned as patients without depressive symptomatology P(−) (see Fig. 2). All patients with a valid criteria 1 (no psychological disorder) or 4 (no psychological disorder likely) served as a pool for the random selection of the patients without psychosocial comorbidity. Of the patients without psychosocial comorbidities (valid criteria 1 or 4), 80% were randomly selected to remain in the study as P(−) patients. Even though the algorithm to select P(+) patients was established to identify patients with depression, the PPV of 68.8% does not allow an immediate diagnosis of a depressive disorder. Not only were patients with diagnosable depression selected, but also patients with relevant symptoms of depression (not completely fulfilling the diagnostic criteria for depression). Therefore, P(+) patients are defined as patients with a diagnosable depression or depressive symptoms and will be referred to as “patients with depressive symptomatology.” In the next step the GP of each patient, who remained in the study after random selection, was interviewed.

### GP telephone interview

The telephone interview with the patients’ GPs was scheduled for three weeks after the baseline questionnaire was sent out to the patients. The interview started with questions regarding the GP’s sociodemographic data and specifications, as well as practice details. In the second part, the GPs were interviewed regarding the comorbidities of each patient. This included asking the GP about a valid HF diagnosis, the New York Heart Association Functional Classification (NYHA class) and comorbidities of the Charlson Index [32], followed by the question: “Does the patient currently display a depressive symptomatology?” If this question was answered with “yes,” the GP was asked whether he or she judges the symptoms as a clinical depression or as sub-threshold symptoms and what measures he or she had taken thus far to treat said symptoms. Then the GP was asked to name the Codes of the *International Statistical Classification of Diseases and Related Health Problems (10th version)* (ICD-Codes) [33] of all further chronic and acute diseases the patient had at the time of the interview. In the next step, all conditions of the Charlson Index were transformed into three-digit-ICD-Codes. The three-digit-ICD-Codes of both sources (Charlson Index and the ICD-Codes stated by the GP) were grouped into 42 comorbidity groups based on the MultiCare list of chronic conditions [34]. Four of the MultiCare conditions were excluded as they presented inclusion or exclusion criteria for this study (“Dementia” was an exclusion criterion in this study, “Cardiac insufficiency” was a required criterion (all patients had a diagnosis of HF), and “Depression” as well as “Anxiety” were directly measured by the patient questionnaire). The number of comorbidities according to the MultiCare list of chronic conditions was summed up to create the comorbidity score.

### Analysis of awareness rates and associated factors

The patients were grouped for analysis according to the information from the patient questionnaires (P[+] or P[−]) and the GP interviews (see Table 1 and Figs. 1 and 2).

**Table 1:** Patient groups for analysis

		GP judgement: depressive symptomatology (GP interview)	
		yes	no
Depressive symptomatology according to algorithm (patient questionnaire)	yes	P(+/+)	P(+/−)
	no	P(−/+)	P(−/−)

*P* patients, the first ± refers to algorithm defined depressive symptomatology, the second ± to the GP statement of the patient’s depressive symptomatology

Based on all patients with depressive symptomatology P(+), the rates of those whose GP was aware of the patients’ depressive symptomatology P(+/+) were calculated.

Additionally, the group P(−/+) was further investigated: This combination may result from patients with a diagnosis of depressive symptomatology who received successful medical treatment and are currently in remission. Those patients would not screen positive, but the GP may be aware of a depressive disorder. This might result in a seemingly false positive diagnosis but can be differentiated in the GPs’ statements (“history of depression” instead of “current depressive symptoms” or “current depression”). Taking this into account and following the recommendation of Joling et al. to consider antidepressant prescriptions [35], an alternative awareness-rate was calculated, which defines all patients as P(+) who screened positive for depressive symptomatology or who were taking prescription antidepressants. Accordingly, the definition of awareness was expanded to include GP ratings of “depressive symptoms”, “depression” or “history of depression”.

The baseline data was analyzed with SPSS Version 20. Descriptive analyses were performed to compare the groups. Logistic regression analyses were calculated to investigate factors associated with the GPs’ (un-) awareness of their patients’ depressive symptoms. Significant associations were assumed when *p* ≤ 0.05. No data imputation strategies were applied.

### Ethics approval

The study was conducted in compliance with the Declaration of Helsinki. The study protocol was approved by the local Ethics Committees (Main study: Medical Association of Hamburg, Approval No. PV3889; Ethics Committee of the Medical Faculty of the University of Würzburg, Approval No. 125/12. Substudy: Ethics Committee at the University of Göttingen Medical Center, Approval No. 19/8/11).

## Results

### Sampling frame

Of 13,830 HF patients invited by their GPs, 4909 patients consented to participate in the study and sent back a baseline questionnaire (see Fig. 1). After random deselection and exclusion of patients not fulfilling the inclusion criteria, 3224 patients were included into the analysis. The patient characteristics are displayed in Table 2. The 3224 patients were treated by 272 GPs of which 73.9% were male. They had a mean of 15.2 (SD 9.4) years of professional experience. A mean of 2.1 GPs worked in each practice (SD 1.1) and 18.8% had further education in psychology/psychiatry (three had additionally specialized in psychiatry, neurology or psychosomatics; forty-five had further training in psychotherapy, psychosomatics or psychology; three had both, the additional specialization as well as further psychotherapeutic training). Telephone interviews with the GPs were conducted a mean of 0.9 (±0.8) months after the patients filled out the questionnaire.

**Table 2:** Patient characteristics

	Patients screened positive for depressive symptomatology			Patients screened negative for depressive symptomatology		
	GP aware of depressive symptoms	GP not aware of depressive symptoms	Total	GP stated no depressive symptoms	GP stated depressive symptoms	Total
	P (+/+)	P (+/−)	P (+)	P (−/−)	P (−/+)	P (−)
Total, N (%)	201 (100%)	373 (100%)	574(100%)	2229 (100%)	421 (100%)	2650 (100%)
Age, mean (SD)	71.9 (11.7)	73.3 (11.4)	72.8 (11.5)	74.4 (9.9)	73.4 (9.5)	74.2 (9.9)
Male gender, N (%)	88 (43.8%)	190 (50.9%)	278 (48.4%)	1272 (57.1%)	178 (42.3%)	1450 (54.7%)
NYHA classification, N (%)						
Class I	30 (14.9%)	60 (16.1%)	90 (15.7%)	576 (25.8%)	104 (24.7%)	680 (25.7%)
Class II	80 (39.8%)	180 (48.3%)	260 (45.3%)	1135 (50.9%)	200 (47.5%)	1335 (50.4%)
Class III	74(36.8%)	110 (29.5%)	184 (32.1%)	440 (19.7%)	97 (23.0%)	537 (20.3%)
Class IV	12 (6.0%)	20 (5.4%)	32 (5.6%)	38 (1.7%)	15 (3.6%)	53 (2.0%)
GP judgement: depressive symptomatology (apparent depression or depressive symptoms), N (%)	201 (100%)	0 (0%)	201 (35.0%)	0 (0%)	421 (100%)	421 (15.9%)
GP judgement: No depressive symptoms	0 (0%)	373 (100%)	373 (65.0%)	2229 (100%)	0 (0%)	2229 (84.1%)
GP judgement: Depressive symptoms	69 (34.3%)	0 (0%)	69 (12.0%)	0 (0%)	195 (46.3%)	195 (7.4%)
GP judgement: Apparent depression	132 (65.7%)	0 (0%)	132 (23.0%)	0 (0%)	226 (53.7%)	226 (8.5%)
GP judgement: History of depression	106 (52.7%)	58 (15.5%)	164 (28.6%)	191 (8.6%)	227 (53.9%)	418 (15.8%)
PHQ-9 Score, mean (SD)	14.3 (4.4)	13.0 (3.5)	13.4 (3.9)	4.0 (3.1)	5.6 (3.4)	4.3 (3.2)
Taking any prescription antidepressant	61 (30.3%)	46 (12.3%)	107 (18.6%)	83 (3.7%)	116 (27.6%)	199 (7.5%)

*P (+)* Patients screened positive for depressive symptomatology, *P (−)* Patients screened negative for depressive symptomatology, P (+/+) patients screened positive for depressive symptomatology and GP stated depressive symptomatology, P (+/−) = Patients screened positive for depressive symptomatology and GP did not state depressive symptomatology, *P (−/−)* Patients screened negative for depressive symptomatology and GP did not state depressive symptomatology, *P (−/+)* Patients screened negative for depressive symptomatology and GP stated depressive symptomatology.

### Rates of depressive symptomatology and GPs’ awareness of depressive symptomatology

Of the 4909 incoming questionnaires, 690 screened positive for depressive symptomatology P (+). This is an overall rate of depressive symptomatology of 14.1%. Of the 690 patients, 574 were eligible for further analysis (see Fig. 1 group P (+)). Table 2 displays the number and characteristics of patients in each of the four groups P(+/+), P(+/−), P(−/−) and P (−/+) in detail. The GPs were aware of their patients’ depressive symptomatology (sensitivity) in 35.0% of all patients who screened positive for depressive symptomatology, while the GPs were not aware of the depressive symptomatology of 65.0% of the positively screened patients (see Table 2). The GPs stated no depressive symptomatology (specificity) in 84.1% of all patients who screened negative for depressive symptomatology.

Noteworthy, GPs stated a depressive symptomatology in 15.9% of the P(−) patients in the telephone interviews, even though the patients did not screen positive for depressive symptomatology P (−/+).

These patients had a relatively high rate of taking prescription antidepressants (27.6%) and the alternative awareness rate (see methods section for definition) was calculated. This alternative awareness rate was considerably higher than the originally estimated awareness rate: 407 of 772 HF patients (50.7%) were identified as suffering from depressive symptomatology ﻿by the GP.

### Factors associated with GPs’ awareness of depressive symptomatology

The results of the logistic regression (dependent variable: *awareness of depressive symptomatology)* are displayed in Table 3. The model includes both patient and GP characteristics. The frequently used PHQ-9 was included in the models for depressive symptomatology to maintain comparability with other studies. Factors associated with the awareness of depressive symptomatology were: higher patient education levels, a history of depression known to the GP, GP-consultation due to emotional distress within the last 6 months, a higher frequency of GP contacts within the last 6 months, a higher NYHA class and a more severe depressive symptomatology. None of the GP characteristics were significantly associated with the awareness of depressive symptomatology in HF patients.

**Table 3:** Logistic regression with endpoint: awareness of depressive symptomatology (patient and GP characteristics)

	Patient and GP characteristics	
Variable	*p*-value	Odds-Ratio [CI]
***Patient characteristics***		
Age	0.970	1.000 [0.975; 1.024]
Sex (male)	0.430	1.226 [0.740; 2.031]
**Education (CASMIN) (Primary)**	**0.001**	
**Secondary**	**0.001**	**2.782 [1.542; 5.017]**
**Tertiary**	**0.021**	**3.599 [1.217; 10.642]**
Employment (not employed)	0.517	1.390 [0.513; 3.767]
Health insurance (statutory)	0.973	
Private	0.944	0.955 [0.270; 3.376]
Social security office	0.823	0.714 [0.037; 13.824]
Living situation (living alone)	0.651	
together with others in private household	0.788	1.082 [0.609; 1.924]
living in an institution	0.354	1.812 [0.516; 6.367]
**NYHA Class III-IV (reference Class I-II)**	**<0.001**	**2.518 [1.498; 4.231]**
Comorbidity score	0.379	0.958 [0.870; 1.054]
**PHQ-9**	**0.027**	**1.075 [1.008; 1.147]**
**History of Depression**	**<0.001**	**6.181 [3.562; 10.723]**
**Number of GP contacts (last 6 months)**	**0.028**	**1.036 [1.004; 1.070]**
**GP-consultations due to emotional distress within the last 6 months (no)**	**<0.001**	**3.185 [1.930; 5.256]**
Familiarity of patient	0.400	1.062 [0.923; 1.221]
Perceived social support (ESSI)	0.764	1.007 [0.964; 1.051]
***GP characteristics***		
Sex (male)	0.826	1.067 [0.598; 1.905]
Years of work experience	0.937	0.999 [0.974; 1.025]
Number of GPs practicing in the medical practice	0.942	1.008 [0.805; 1.263]
Additional qualification in psychology/psychiatry (no)	0.442	0.787 [0.427; 1.450]
Constant	0.002	0.017

*Bold letters* significant association, *CI* 95% Confidence Interval, *N* = 417, R^2^ = 0.335

## Discussion

### Main results

In this observational study, depressive symptomatology was found in 14.1% of primary care patients with HF, when applying the algorithm established in a previous substudy [30]. GPs were aware of the depressive symptomatology of 35% of their patients with a positive depression screening, which is lower than hypothesized.

Factors with the strongest association with the awareness of depressive symptomatology were: higher patient education levels (OR 3.6 for tertiary education compared to primary education), a history of depression known to the GP (OR 6.2), GP-consultation due to emotional distress within the last 6 months (OR 3.2), and a higher NYHA class (OR 2.5 for NYHA class III-IV compared to I-II). Contrary to our hypothesis, none of the investigated GP-based factors were significantly associated with the GP’s awareness of depressive symptomatology.

### Low rates of depressive symptomatology

The rates of depressive symptomatology in our present study are on the lower end of rates found in other studies. A systematic review [36] found rates of comorbid depression between 13% and 48% in outpatients with HF, depending on the assessment tools used and the HF severity. We explain the low rates in our study as due in part to the chosen screening algorithm and recruitment strategy. The algorithm adjusts for overlapping symptoms between depression and HF and, therefore, is more conservative than screening instruments with cut-off values for the general population (as administered in other studies, like in Cully et al.’s study [9]). This might lead to an over-estimation of depression rates in HF patients in other studies. Furthermore, HF patients with depressive symptomatology might be less likely to respond to an invitation letter from their GP than HF patients without depressive symptomatology. Therefore, we cannot exclude that true rates of depressive symptomatology in HF patients in primary care are slightly higher than the rates we found.

### Low GP awareness rates of depressive symptomatology

The awareness rates of depression in our study are considerably lower than hypothesized. There are several explanations which can be divided into formal (such as the studied population and the definition of awareness/recognition) and practical reasons:

The GPs’ rate of correct diagnoses of depression in HF patients after hospital discharge where higher in the studies by Koenig et al. (63.2%) [19] and Cully et al. (57.5% recognition of depression/anxiety) [9] than the rate found in our study (35%). Both cited studies recruited patients via a hospital and, therefore, probably included more severely ill HF patients with a higher prevalence of depression and more severe symptoms than the patients included in this study. The higher recognition rates in the cited studies compared to the rate in our study may be attributed to the more frequent and more severe depressive disorders as well as a more intense aftercare following hospital discharge. We also showed that the alternative awareness rate in our study rises to 50.7% depending on the definition of depressive symptomatology and the definition of awareness, which then corresponds to the overall recognition rate of 47.3% found by Mitchell et al. in a meta-analysis in primary care patients [18]. Additionally, we assume that the study focus on comorbidity instead of depression better corresponds to the clinical practice and prevented artificially elevated awareness rates. Therefore, the rates in the present study might be more accurate than in studies where the focus on depression was known to the GP.

There are several possible practical reasons for the low awareness rates found in our present study: Firstly, symptoms of depression might not be presented to the GP. This corresponds with our finding that consulting the GP due to emotional distress raised the GP’s chance of awareness. Secondly, in everyday practice, concurrent issues such as the treatment of somatic problems might be considered more relevant at the time. Thirdly, the symptom overlap between HF and depression, as well as, the volatility of depressive symptoms complicates their recognition. Fourthly, we hypothesize that GPs do not diagnose depression before they initiate a treatment or before there is a need for action on their part. Therefore, they might have answered the question if the patient displayed symptoms of depression with “no” even though they knew about them. This hypothesis is supported by Cully et al., who showed that 92.3% of all HF patients diagnosed with depression and/or anxiety had received corresponding treatment [9]. Further, studies in primary care have shown that GPs’ diagnoses of depression differ from psychiatrically based diagnostic criteria such as ICD-10 [37, 38] and DSM IV [39]. For example, GPs tend to attribute depression mainly to a reaction to certain circumstances [37] [39], a “grey area” [38], and are reluctant to diagnose depression, if the circumstances seem to explain a patient’s depressive symptoms. However, even if symptoms can be explained by circumstances, a patient might suffer from an actual depressive episode and may benefit from treatment. Thus, there are probably HF patients who would benefit from depression treatment, but don’t receive treatment because the GP is not aware of their depressive symptomatology.

### Factors associated with (un-)awareness of depressive symptomatology

In this study we found that a history of depression known to the GP and consulting the GP due to emotional distress significantly raised the likelihood of the GP being aware of depression. This is in line with earlier research [19, 40–42]. Further studies showed that the frequency of GP consultations [40, 41] and the severity of depressive symptomatology [9, 42] was significantly associated with GP awareness, which was also confirmed in our study.

Furthermore, we found that the GPs’ awareness of depressive symptomatology was higher in patients with NYHA classes III-IV than patients in NYHA classes I-II, when controlling for the severity of depressive symptomatology. Cully et al. did not find an association between a patient’s NYHA class and the GP’s recognition of depression (but did not include NYHA class I patients) [9]. One explanation for the unexpected finding could be that patients with a higher NYHA class are known to have more severe depressive symptoms [5] and, therefore, are also more likely to present depressive symptoms during the GP consultation than patients with less severe HF and fewer coexisting depressive symptoms.

We did not find a significant association between a GP’s awareness of depressive symptomatology and a patient’s age or sex; but there was a positive association with patients’ education levels. Findings concerning the influence of age, sex and education are inconsistent [9, 19, 40, 42]. However, in our study, patients with primary education were found to have a lower chance of their GPs being aware of their depressive symptomatology. Therefore, GPs should pay special attention to this group of HF patients.

Contrary to our hypothesis, we found no significant associations between a GP’s awareness of depressive symptomatology and any of the GP-based factors: gender, years of residency, training in psychology/psychiatry, or number of GPs practicing in the physician’s practice. This corresponds with the findings of Piek et al. and Wittchen et al. who found no association between the recognition of depression and the factors: GP gender, years of GP-experience, training in depression or in psychiatry in the past year [40] or the number of depression-specific continuing education courses taken by the GP [42]. The number of GPs practicing in the physician’s practice could also be interpreted as a proxy for practice size and was not a significant predictor of a GP’s awareness of depressive symptomatology in our study. This is in line with the review by Mitchell and Rao [18].

### Strengths and limitations

In contrast to other studies on the recognition of depression [19], our study included patients with and without depressive symptomatology and asked about a variety of patients’ comorbidities without revealing a specific focus on depression, a potential bias in other studies. Therefore, the present study focused on the GP’s statement as to whether or not a patient displayed depressive symptomatology (in this case the sum of medical record data and GP memory, which is interpreted as a GP’s awareness of a patient’s depressive symptomatology), rather than investigating whether or not the GP recognized it. In accordance, the GP was asked whether the patient displays depressive symptomatology and not if he/she would diagnose a depressive disorder. These choices have been made to reflect everyday practice as accurately as possible and the term “awareness” instead of “recognition” of depressive symptomatology was chosen. Furthermore, a sub-study developed an algorithm of optimal cut off values in self-administered depression instruments in a different cohort of patients with HF, to consider the symptom overlap between depression and HF.

However, the study also has some limitations. One limitation is that, although created especially for HF patients, our screening algorithm has a PPV of 69%. Thus, 31% of the patients who screened positive for depressive symptomatology do not have a current depressive disorder. We addressed this limitation by asking the GP, if the patient displays depressive symptomatology instead of asking if the patient meets the criteria for a diagnosis of depression. It can be assumed that all positively screened patients displayed some depressive symptomatology, even if it was not severe enough to be diagnosed as a depressive disorder. However, GPs should be aware of these sub threshold symptoms, because they have been found prognostically relevant, even without fulfilling a formal diagnosis of depression [43]. Also, false-positive rates of depression awareness might actually reflect depressive patients with a successful depression treatment, who are in remission and, therefore, are no longer identified as depressed by our algorithm. To address this limitation, we calculated alternative awareness rates of depressive symptomatology and also present data for the groups P (−/−) and P (−/+). Further, the outcome criterion of GP awareness is derived from the GP’s statement whether or not the patient displays depressive symptomatology. However, we cannot exclude that a GP might be aware of a patient’s depressive symptomatology, but for some reason (e.g. symptoms are not judged as severe enough to be stated) did not state them. Furthermore, the method of telephone interview carries the risk of recall errors. Therefore, true awareness rates might be higher than reported. Lastly the GP (6.6%) and patient (35.5%) response rates were lower than expected. This might have led to an underrepresentation of GPs with high workloads as well as patients severely affected by HF or depression.

### Implications for practice

According to our results, GPs are more likely to miss depressive symptomatology in patients without a known history of depression, who did not consult their GP because of emotional distress, and those with lower levels of education. The awareness rates could be improved, if GPs encourage their HF patients to address emotional distress and held detailed medical interviews including a patient’s history of depression. This remains to be proven in interventional studies. The recommendation to routinely apply screening instruments in HF patients remains questionable as the evidence of its benefit still remains to be provided [16]. Furthermore, it involves the risk of increased false-positive recognition rates [18, 44] and, thus, inappropriate therapies or overtreatment. Therefore, screening instruments for depression in primary care patients with HF should only be applied, if a GP is unsure about a patient’s depression after a thorough examination. Simple screening questions for emotional distress and past depression asked routinely during the medical interview might be preferable, especially for patients with lower education levels. The effect of this mini-intervention on the GP’s awareness of depressive symptomatology in patients with HF remains to be investigated.

The comparably low awareness rates are probably not based on GPs’ lacking knowledge of diagnostic criteria, because, in the present study as well as in a randomized controlled trial [45], GPs’ further psychological education did not increase the rate of awareness/recognition of depression. Instead, there is evidence that GPs view the psychiatry-based criteria as inappropriate for general practice [39].

### Implications for research

The definition of awareness or recognition rates has a great impact on the percentage of recognized patients. GPs seem to have their own diagnostic criteria (including gut feeling) which differ from the psychiatric criteria [38]. When investigating recognition rates these aspects need to be considered in the definition of “recognition” and in the choice of criteria used to identify patients with depression.

Koenig et al. found that hospital physicians and GPs were more likely to identify depression warranting treatment in HF patients whose depression persisted at least 12 weeks [19]. This is a finding which might support the sensitivity of GP rating. It remains to be investigated to what extent the psychiatric criteria are a better or worse indicator for predicting the future quality of life and prognosis of HF patients, and to what extent this reveals a need for action to increase the awareness rates of depressive symptomatology in HF patients in primary care. Therefore, low recognition rates in primary care as judged by psychiatry-based screening instruments need to be carefully investigated regarding patient relevant outcomes.

## Conclusions

Many aspects, including the definition of awareness and practical issues in primary care, may have contributed to unexpected low GP awareness rates of depressive symptomatology in HF patients in primary care. The awareness rates might rise, if GPs encourage their patients to talk about emotional distress, held detailed medical interviews including a patient’s history of depression and pay special attention to HF patients with lower education levels. However, it remains to be investigated whether GP judgement is a better or worse indicator of the future prognosis and quality of life in HF patients compared to psychiatry-based diagnostic criteria. It also needs to be investigated to what extent this information induces a need for action.

## Abbreviations

GP: General Practitioner
HF: Chronic heart failure
ICD-Codes: Codes of the International Statistical Classification of Diseases and Related Health Problems by the WHO
NPV: Negative predictive value
NYHA class: New York Heart Association Functional Classification
PPV: Positive predictive value
